# Unlocking the potential of PubMed Central supplementary data files

**DOI:** 10.1093/bioadv/vbaf155

**Published:** 2025-06-27

**Authors:** Julien Gobeill, Déborah Caucheteur, Alexandre Flament, Pierre-André Michel, Anaïs Mottaz, Emilie Pasche, Patrick Ruch

**Affiliations:** SIB Text Mining Group, Swiss Institute of Bioinformatics, Geneva 1206, Switzerland; BiTeM Group, Information Sciences, HES-SO/HEG Geneva, Carouge 1227, Switzerland; SIB Text Mining Group, Swiss Institute of Bioinformatics, Geneva 1206, Switzerland; BiTeM Group, Information Sciences, HES-SO/HEG Geneva, Carouge 1227, Switzerland; SIB Text Mining Group, Swiss Institute of Bioinformatics, Geneva 1206, Switzerland; BiTeM Group, Information Sciences, HES-SO/HEG Geneva, Carouge 1227, Switzerland; SIB Text Mining Group, Swiss Institute of Bioinformatics, Geneva 1206, Switzerland; BiTeM Group, Information Sciences, HES-SO/HEG Geneva, Carouge 1227, Switzerland; SIB Text Mining Group, Swiss Institute of Bioinformatics, Geneva 1206, Switzerland; BiTeM Group, Information Sciences, HES-SO/HEG Geneva, Carouge 1227, Switzerland; SIB Text Mining Group, Swiss Institute of Bioinformatics, Geneva 1206, Switzerland; BiTeM Group, Information Sciences, HES-SO/HEG Geneva, Carouge 1227, Switzerland; SIB Text Mining Group, Swiss Institute of Bioinformatics, Geneva 1206, Switzerland; BiTeM Group, Information Sciences, HES-SO/HEG Geneva, Carouge 1227, Switzerland

## Abstract

**Motivation:**

Biocuration workflows often rely on comprehensive literature searches for specific biological entities. However, standard search engines such as MEDLINE and PubMed Central provide an incomplete picture of the scientific literature because they do not index the increasing amount of valuable information published in supplementary data files. Over two years, we addressed this gap by systematically extracting text from a large proportion (85%) of these files, resulting in 35 million searchable documents. To assess the information gain provided by supplementary data files beyond the manuscripts, we searched both for mentions of dozens of Global Core Biodata Resources (GCBRs), which are fundamental biological databases essential for the life sciences. We searched for mentions of GCBR names and accession numbers, which uniquely identify biological entities within these resources.

**Results:**

The recall gain from using the supplementary data files to search for articles mentioning resource names is 6%. In addition, 97% of all accession numbers identified were published in the supplementary data files, highlighting their increasing importance for highly specific topics or curation pipelines. We show that the number of accession numbers published in the supplementary data files is increasing year on year, but that 87% of these are published in Excel files. This format facilitates human readability and accessibility, but severely limits machine reusability and interoperability. We therefore discuss alternative and complementary approaches to the publication of research data.

**Availability and implementation:**

All extracted data are accessible and searchable as a collection on the BiodiversityPMC platform (https://biodiversitypmc.sibils.org/).

## 1 Introduction

MEDLINE and PubMed Central (PMC) are well organized big data, providing access to vast biomedical literature: PMC offers free full-text search and access to research articles, complementing the bibliographic details and abstracts available in MEDLINE (Delamotte and Smith 2001). Both collections and their search engines play crucial roles in bio curation workflow for linking biological databases with literature (Bourne et al. 2006), enhanced or complemented by text mining solutions (Névéol 2012, Hirschman *et al.* 2012). Concerning the comparative utility of abstracts and full texts, numerous studies have demonstrated the enhanced information retrieval potential of full-text articles compared to abstracts alone (Lin 2009, Blake 2010, Westergaard *et al.* 2018).

Yet, search engines in MEDLINE and PubMed Central provide an incomplete picture of the scientific literature. Beyond the abstract and the full-text, publications are often accompanied by so far “dark data”: the supplementary data files, which contain valuable information that supports the article but are not included in the core manuscript. These supplementary data files can contain detailed methods, additional results, raw data tables, or extended figures (Ramstrand *et al.* 2020, Boll 2007). But they are highly heterogeneous (images, documents, spreadsheets, code…), and often stored in proprietary formats (e.g. Word, Excel…). This vast amount of valuable information collectively forms a significant portion of scientific knowledge and is critical for scientific progress (Howe *et al.* 2008, Ferguson *et al.* 2014). However, the supplementary data files are currently beyond the scope of literature search, as they are not carefully indexed and stored. They become nearly invisible to scientists, and more likely to remain underutilized or eventually lost (Heidorn 2008). Furthermore, concerns have been raised (Greenbaum *et al.* 2017) about the need to improve the structure of supplementary files to better facilitate the application of the FAIR principles (Wilkinson *et al.* 2016): findability, accessibility, interoperability and reproducibility.

While the existing literature reveals limited research on this topic, some studies showed that supplementary data files could enhance the impact of information retrieval, by providing valuable information beyond what is available in MEDLINE and PMC (Kafkas *et al.* 2015, Jimeno and Verspoor 2014, Naderi *et al.* 2022, Pasche *et al.* 2023). However, these studies have been conducted on samples of PMC, and/or focused on a limited number of file types. Recently, BioStudies (Sarkans *et al.* 2018) serves as a repository that organizes and provides access to supplementary data files associated with life sciences studies, including those from Europe PMC. Yet, BioStudies does not directly extract text from supplementary data files, and therefore does not make them findable via a search engine.

During two years, we have systematically downloaded all PMC supplementary data files and extracted the text from a large proportion of them. Extracted texts were then indexed and made available as a new collection in our Swiss Institute of Bioinformatics Literature Services (SIBiLS), accessible via RESTFUL APIs for searching and fetching, and a Web search engine (Gobeill *et al.* 2020). To assess the information gain provided by the supplementary data files, we exploited the Global Core Biodata Resources (GCBR), a set of fundamental biological data repositories that are crucial for life science research worldwide (Cook and Cochrane 2023). For the 52 resources included in 2024, we searched for all references to their names in the supplementary data files, and compared them with references in MEDLINE and PMC. We then repeated this work with accession numbers, which are unique and stable identifiers assigned to specific entries within GCBRs, e.g. “GO: 0005739” which is the accession number in the Gene Ontology for the concept “mitochondrion.” Matching an accession number within a text strongly suggests the presence of information about a particular biological entity.

In this article, we describe the methods used to extract text from these heterogeneous files, and to recognize GCBR concepts in text. We show that the supplementary data files contain a massive amount of valuable keywords and accession numbers that are not present in the manuscript of the article, and would otherwise be missed by a search in PubMed or PMC. We also show how this data sharing is evolving, and what file formats are being used by authors.

## 2 Methods

The experiments reported in this article were performed in July 2024 using 37.4 million bibliographic references from MEDLINE, 6.2 million manuscripts from the PMC Open Access (OA) set, and their associated supplementary data files. In this section, we first present SIBiLS, which already contains the MEDLINE and PMC collections, and into which we have integrated the supplementary data files collection. We then describe how we extracted the text from these supplementary data files. Finally, we describe how we searched for GCBR names and accession numbers in all these collections.

### 2.1 Integration of supplementary data files in SIBiLS

**Figure 1.**
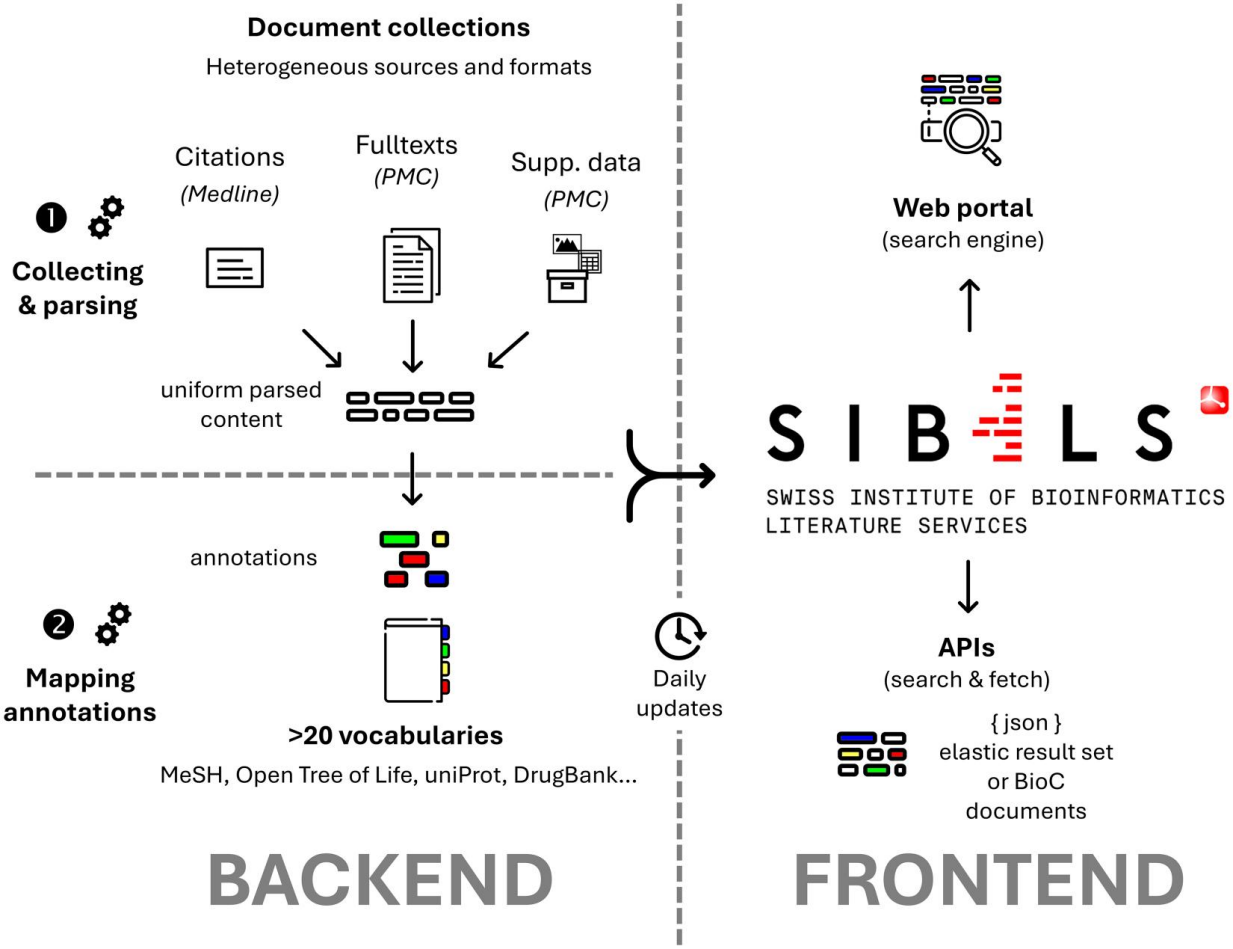
General workflow of SIBiLS. Other collections not represented are also present in SIBiLS, such as Plazi treatments, PMC authors manuscripts, ClinicalTrials, and articles from several biodiversity journals.

The Swiss Institute of Bioinformatics Literature Services (SIBiLS) is a resource for personalized Information Retrieval of biomedical literature. SIBiLS collects and updates several collections on a daily basis. All documents are parsed and then semantically enriched by automatic annotation using about thirty reference controlled vocabularies, such as Medical Subject Headings (MeSH), Open Tree of Life or Drug-Bank. Users can search the collection using the classic SIBiLS Web search engine, or by submitting their own personalized query (using Elastic Query Language) via the RESTFUL APIs. Users can also retrieve a set of documents along with their annotations, in JSON BioC format, thanks to the fetch API. The Fig. 1 illustrates the general workflow of SIBiLS.

MEDLINE and PMC collections are both collected using the National Library of Medicine (NLM) ftp server (ftp.ncbi.nlm.nih.gov). The NLM updates these collections daily by providing tar.gz files containing one XML file for each document added or updated. For the last two years, SIBiLS has also indexed a collection of supplementary data files from a subset of PMC articles that were presumably relevant to genetic variants, obtained using a Boolean search query (Pasche 2023). In parallel, we extracted the text of supplementary data files from the entire PMC collection, which have been fully available in SIBiLS since July 2024. In this collection, each supplementary data file is considered as a document. A unique identifier is created by concatenating the PMCID (PMC article identifier) and the filename (e.g. PMC11065014_media-1.pdf).

### 2.2 Text extraction from the supplementary data files

For each article in the PMC OA set, a tar.gz archive containing a set of files (both related to the manuscript and the supplementary data files) is available on the NLM ftp server. Among these files, we have excluded the cXML files that contain the manuscript, and whose information is therefore already in the PMC collection. We also excluded PDF files with the same name as the nXML, as this is the PDF version of the manuscript. We processed all JPG files, but we distinguished between figures that are part of the manuscript (their names are indicated in the nXML) and those that are really supplementary data. Finally, we excluded GIF images, as they are either miniature versions of a figure in JPG (for Web printing) or equation symbols. This left 36.2M files for 6.2M articles, with more than 1200 different file types.

We then used several methods to extract the text from different types of files:

- for JPG files, we used a local API (https://ocrweb.text-analytics.ch/) based on Tesseract (Smith 2007), a popular Optical Character Recognition (OCR) software with proven accuracy (Tafti *et al.* 2016). Our API optimizes the OCR by applying rotations to the image to enable vertical text to be recognized.
- For PDF, we used the Python module PyPDF2 (https://pypi.org/project/PyPDF2/). This module extracts text stored as actual characters (i.e. searchable and selectable).
- for tables (xlsx, xls, and csv), we used a local extractor developed by (Naderi *et al.* 2022) based on the Python module pandas (McKinney 2011).
- for Word documents (doc, docx), we used the Python modules textract and doc2txt (https://textract.readthedocs.io/en/stable/, https://pypi.org/project/docx2txt/).
- for HTML and XML, we extracted the text using the Python module BeautifulSoup (https://pypi.org/project/beautifulsoup4/).

It took two years to download and process all the supplementary data, with parallel programs. Table 1 shows the twenty most common file types in the supplementary data files, which represent 98% of all the files.

**Table 1.** For each file type, the table shows the number of files present in the supplementary data, and the equivalent percentage.^a^

File type	# files	%	Success rate	Average length
jpg	5 707 606	48.62%	100%	341
pdf	1 658 182	14.13%	99.9%	16 947
docx	1 079 109	9.19%	99.9%	8164
tif	782 252	6.66%		
xlsx	681 736	5.81%	99.7%	39 015
txt	313 630	2.67%	99.4%	17 552
zip	310 209	2.64%		
Doc	215 628	1.84%	97.4%	8288
mp4	188 258	1.60%		
xls	124 083	1.06%	95.7%	45 019
avi	60 747	0.52%		
xml	57 541	0.49%	100%	14 810
html	52 622	0.45%	99.8%	7037
mov	48 364	0.41%		
png	48 030	0.41%		
csv	40 495	0.34%	93.8%	112 296
flv	40 045	0.34%		
eps	39 900	0.34%		
cif	39 860	0.34%		
webm	37 475	0.32%		

a The success rate is defined as percentage of files for which the text extraction succeeded, and the average length is the average number of characters per file.

A further 24 491 367 JPG files are figures that are part of the manuscript. They are not included in the previous table, but we have also processed them to include their text in our supplementary data files index (as their text is not indexed and therefore not searchable in the PMC collection).

In terms of supplementary data files alone, we thus extracted text from 84.6% of the files. The initial design of our text extraction pipeline was based on the assumption that JPG would be the dominant format for figures and images in supplementary data files; however, we later observed that TIFF and PNG formats represented a non-negligible 7% of the image data, which were not processed. The zip files (2.64%) are currently being processed but are not yet available at the time of the study. Finally, more than 3% of the files are multimedia videos (MP4, AVI, MOV, FLV, and WebM), which seem more difficult for us to process.

Extraction success rates are nearly 100%, except for older Microsoft doc (97.4%) and XLS (95.7%) formats, where some files present issues even when opened manually with the correct software, and for CSV files (93.8%), which may contain formatting errors or problematic encoding. For JPG, 23% of successfully extracted files have no extracted text. For the rest, output quality may be low depending on the image resolution. Additionally, while text from doc and PDF files is extracted, some of these files consist of scanned images, and OCR is not applied to them.

### 2.3 Search for references to global core biodata resources

In July 2024, there were 52 resources listed as GCBR. We took the 52 names and searched for them in SIBiLS, first in the MEDLINE collection (title + abstract), then in the PMC collection (full text), and finally in the supplementary data files collection (text extracted from the files).

The resources names were searched thanks to regular expressions using lowercasing and word boundaries, ensuring, for example, that Rhea is not recognized in the word “spearhead” (>3000 results in PMC). We considered only articles published from 2000. For MEDLINE, we used the PMCID as the unique identifier of the article when it was available, to avoid counting the same article twice if the mention was in both the abstract (MEDLINE and PMC) and the full text (PMC). For 72% of the retrieved MEDLINE citations, the article was available in PMC. For supplementary data files, we have also used the article PMCID to avoid counting the same article twice if the mention was in both the abstract or full text (MEDLINE and PMC) and the supplementary data files. Four resources were excluded from the results after a manual scan of a sample of 1000 sentences containing a mention, since they caused between 50% and 75% of False Positives: BRENDA and SILVA (which were confused with first names or surnames), CATH (confused with the abbreviation of catheter, e.g. “cath lab”), and STRING (confused with the common noun “string”).

For the accession numbers, we also used regular expressions, collected in the Identifiers.org Central Registry, a service that provides a centralized directory of Compact Identifiers for biodata resources (https://registry.identifiers.org/). Only 30 of the 52 resources were found in the registry. These are listed in Table 2. For each resource, we exploited its “Local Unique Identifier pattern,” a regular expression (e.g. r”GO:\d{7]” for the Gene Ontology). Pre-experiments showed that many common nouns were identified as accession numbers because certain regular expressions were too general, such as r”[23456789BCDFGHJKLMNPQRSTVWXYZ]{1,6}” for the Catalog of Life. The same problem occurred with UniProt identifiers. We then decided to force the presence of the resource (e.g. “col:4QHKG”, “UniProt:Q8VRN4”) for those problematic regular expressions. This probably led to missed identifiers, but solved the problem of False Positives in the samples. We also used lowercasing for regular expressions.

**Table 2.** The table shows the number of accession numbers identified for each resource, depending on whether we searched in MEDLINE only, or added PMC, or added the supplementary data files.^a^

Global core biodata resource	MEDLINE	MEDLINE + PMC	MEDLINE + PMC + suppdata	% only in suppdata
BacDive	0	3	3	0%
Bgee	15	35 976	3 271 115	99%
BRENDA	0	9	255	96%
Catalogue of Life	6	1947	2590	25%
CATH	0	39	75	48%
Cellosaurus	7	67 365	88 699	24%
ChEBI	1	5883	70 320	92%
ChEMBL	203	32 832	306 232	89%
Clinical interpretation of variants in cancer	0	0	0	
Ensembl	435	69 678	4 046 100	98%
FlyBase	36	60 243	3 804 092	98%
Gene ontology	873	1 262 847	29 948 969	96%
GWAS catalog	40	19 659	267 619	93%
Human protein atlas	281	219 672	18 131 116	99%
IMEx	0	6	3897	100%
InterPro	48	70 986	1 989 334	96%
LIPID MAPS	0	0	168	100%
PANTHER	0	81	6078	99%
Pharmacogenomics knowledgebase	0	1398	12 139	88%
PomBase	0	33	33	0%
Protein data bank	2	255	335	24%
ProteomeXchange consortium	0	3	3	0%
Rat genome database	4	312	15 516	98%
Reactome	85	37 527	710 763	95%
Rhea	0	588	6197	91%
Saccharomyces genome database	0	579	6675	91%
STRING	1	99	2443	96%
UniProt	1	2,547	43 516	94%
WormBase	0	9	23	61%
Zebrafish information network	3	8964	194 267	95%

TOTAL	2041	1 899 540	62 928 572	97%

a The last column is the percentage of accession numbers exclusively found in the supplementary data files.

We used different counting strategies for resource names and accession numbers, depending on our specific objectives. For resource names, we were interested in the number of articles that cited a resource. Therefore, we counted a citation only once per article, regardless of whether it appeared in the manuscript, supplementary data files, or both. For accession numbers, however, our aim was to quantify the number of additional mentions in the supplementary data files. Therefore, we counted each identified accession number to directly compare its prevalence between the manuscript and the supplementary data files. Articles in MEDLINE and PMC include both title and abstract, but we were careful not to count accession numbers twice.

It is crucial to note that the primary focus of this study is not to exhaustively identify every mention of GCBRs, but rather to compare the relative amount of information within supplementary data files against within manuscripts. For this purpose, we assume that we can rely on simple methodological approach, prioritizing high precision (minimizing false positives) over maximal recall (capturing all possible in-stances).

## 3 Results

### 3.1 The growing amount of available supplementary data files

**Figure 2.**
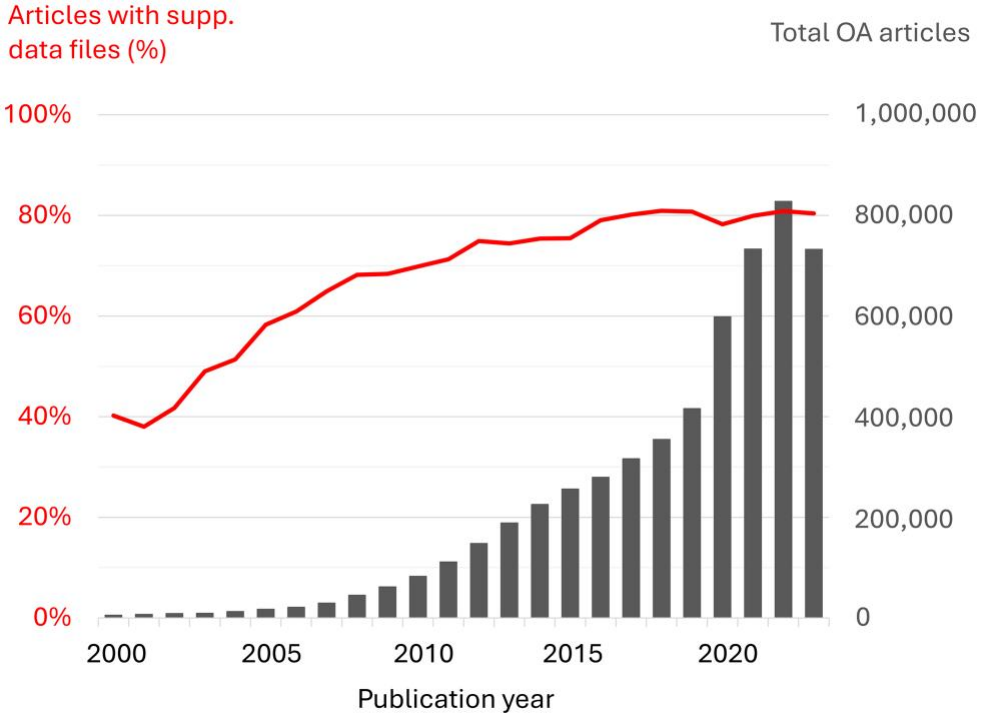
Evolution of PMC OA articles and supplementary data files. In grey, numbers of articles in the PMC Open Access set, per publication year. In red, percentage of articles with supplementary data files.

We first observed the evolution of the number of supplementary data files according to the publication year, which is illustrated in Fig. 2. The number of open access articles is increasing year on year, and the number of articles with supplementary data files has risen from around 40% in the early 2000s to around 80% in the 2020s. This suggests that authors are increasingly including supplementary data to support their findings, possibly due to journal requirements to promote FAIRness, or due to the availability of data offered by the computer generation of large datasets. In the early 2020s, there was a significant rise in the number of OA articles, likely due to the impact of COVID-19 on publication volume, as well as a shift in the publishing landscape driven by Open Access mandates from funding institutions. The reasons for the concomitant slight decrease in the average number of supplementary files have yet to be explained. Finally, the smaller number of articles available for 2023 may be explained by the fact that some are still under publication embargo in mid-2024.

### 3.2 References to GCBR names in supplementary data files

We then report in Table 3 the number of articles with mentions of GCBR names in the three collections available: first by searching MEDLINE only, then MEDLINE and PMC, then MEDLINE and PMC and supplementary data files (abbreviated as supplementary data in the following tables). Finally, we looked at the benefits of using supplementary data files to find articles that mentioned a resource, compared to searching MEDLINE and PMC alone.

**Table 3.** The table shows the number of articles in which the name of each resource was identified, depending on whether we searched in MEDLINE only, or added PMC, or added the supplementary data files.^a^

Global core biodata resource	MEDLINE	MEDLINE + PMC	MEDLINE + PMC + suppl. data	Gain with suppl. data
Alliance of genome resources	23	195	223	+13%
BacDive	6	198	237	+16%
Bio-analytic resource for plant biology	2	92	95	+3%
Bgee	20	245	339	+28%
Catalogue of life	18	403	464	+13%
Cellosaurus	15	835	966	+14%
ChEBI	123	2163	2714	+20%
ChEMBL	824	4317	4722	+9%
Clinical interpretation of variants in cancer	6	97	114	+15%
Clinical genome resource	112	507	576	+12%
DNA data bank of Japan	101	3115	3247	+4%
EcoCyc	83	1222	1432	+15%
Ensembl	1412	55 813	65 574	+15%
Europe PMC	181	412	544	+24%
European nucleotide archive	186	14 046	14 490	+3%
FlyBase	155	6747	7741	+13%
GENCODE	173	12 819	14 746	+13%
Gene ontology	33 637	165 986	170 603	+3%
Global biodiversity information facility	262	3099	3208	+3%
gnomAD	834	15 505	16 710	+7%
Genome sequence archive	16	3563	3619	+2%
GWAS catalog	289	4953	5484	+10%
GXD	54	263	311	+15%
HUGO gene nomenclature committee	81	2362	2611	+10%
Human disease ontology knowledgebase	0	0	0	
Human protein atlas	1648	14 019	14 734	+5%
IMEx	82	994	1234	+19%
InterPro	468	13 490	17 959	+25%
IUPHAR/BPS Guide to PHARMACOLOGY	18	987	1028	+4%
LIPID MAPS	96	2126	2341	+9%
List of Prokaryotic names with standing in nomenclature	20	528	551	+4%
Mouse Genome Database	86	848	1006	+16%
Orphadata Science	0	0	0	
PANTHER	1076	14 254	16 597	+14%
Pharmacogenomics knowledgebase	53	281	294	+4%
Planteome	4	82	92	+11%
PomBase	17	583	685	+15%
Protein data bank	4387	59 620	61 506	+3%
ProteomeXchange consortium	252	13 850	14 108	+2%
Rat genome database	63	653	698	+6%
Reactome	1138	19 954	23 038	+13%
Rhea	360	2280	2542	+10%
Saccharomyces genome database	120	3164	3413	+7%
UCSC genome browser	342	19 395	21 753	+11%
UniProt	2009	72 919	83 125	+12%
VEuPathDB	6	172	194	+11%
WormBase	112	4,095	4680	+13%
Zebrafish information network	23	354	386	+8%

ANY OF THEM	48 849	383 559	407 039	+6%

a The “any of them” line is not the sum, as one article can mention several resources.

Overall, the recall gain using the supplementary data files is 6%, with notable values for Bgee (+28%), InterPro (+25%), and EuropePMC (+24%). In this work, we have taken the names of the resources as given by the Global Biodata Coalition. This led to False Positives, the main sources of which were discarded, but certainly also to False Negatives. For example, “Orphadata science” had no match, whereas “Orphadata” returns over 1000 results in PMC.

Authors may therefore use a resource in their work without mentioning it in the manuscript, but only in the supplementary data files. The impact of these resources is therefore greater than what can be measured by a search in MEDLINE or PMC alone.

### 3.3 References to GCBR accession numbers in supplementary data files

We then report in Table 2 the total number of accession numbers found in the three collections, using the regular expressions collected from identifiers.org for 30 resources: first by considering MEDLINE only, then MEDLINE and PMC, then MEDLINE and PMC and supplementary data files. Finally, we looked at the percentage of accession numbers that were only present in the supplementary data files.

97% of all mentions of accession numbers are found in the supplementary data files. While the recall gain was significant but modest for resource names (+6%), here the number of retrieved documents is multiplied by 33, rising from 1.9 to 62.9 million. The most frequent mentions come from the Gene Ontology, with nearly 30 million references, and Human Protein Atlas, with 18 million. We suspect that the number of UniProt accession numbers contained in the supplementary data files is actually much higher, but it would have been impossible to identify them correctly without the restrictions we imposed by forcing the resource name beforehand (e.g. “UniProt: Q8VRN4”). The only resource without found mentions is Clinical Interpretation of Variants in Cancer (r”civic.vid:\d+”).

It is therefore certain that by searching MEDLINE and PMC alone, a scientist looking for literature on a particular accession number will miss the largest part of references.

For assessing the quality of extracted accession numbers and proving that our assertions were right, we randomly selected 1000 of them, and checked if they were correct, i.e. if they existed in the related biodata resource. The results are presented in Table 4.

**Table 4.** The table shows the number of accession numbers for each resource in our representative sample of 1000, and how many are correct, i.e. they exist in the corresponding resource.

Biodata resource	Example	Accession numbers	
		In sample	Correct
Bgee	FBgn0036915	54	53
ChEBI	chebi: 18034	2	2
ChEMBL	CHEMBL1148970	6	6
Ensembl	ENSG00000176261.11	63	63
FlyBase	FBgn0003074	69	69
Gene ontology	GO: 0005737	485	485
GWAS catalog	GCST90017001	2	2
Human protein atlas	ENSG00000122862	277	277
InterPro	IPR013770	31	31
Pharmacogenomics knowledgebase	PA444801	1	1
Rat genome database	RGD: 69310	1	1
Reactome	R-HSA-2168880	3	3
UniProt	UniProt: Q19724	1	1
Zebrafish information network	ZDB-GENE-030820–3	5	5
		
		1000	999

These results show that the regular expressions provided by identifiers.org are extremely accurate (99.9%). The only spotted error was an incorrect Bgee accession number (FBgn003202), which was actually FBgn0032029 in the original file (a nine-page table in doc format) and was badly extracted. Such tables in Word documents may be challenging to parse, due to lack of clear borders, merged cells, or inconsistent spacing.

### 3.4 Evolution by publication years and file types

Finally, we observed how the number of accession numbers has evolved according to the publication year, and the type of file used, and reported the results in Table 5.

**Table 5.** The table shows the number of accession numbers identified in the supplementary data files, according to the publication year of the article, and the type of file used by authors.

File type	2018	2019	2020	2021	2022	2023
xlsx	3 131 940	4 024 386	4 464 787	6 614 406	7 362 218	6 660 721
xls	415 048	530 603	459 247	469 713	610 805	413 625
csv	161 923	205 441	267 969	405 107	472 132	438 542
txt	230 406	151 358	230 885	229 838	176 937	266 968
pdf	115 001	180 974	243 979	244 976	228 858	198 941
docx	43 176	75 927	128 257	169 618	165 457	105 161
doc	13 802	3 379	2401	9180	2981	1498
xml	4 459	2 179	914	6	9335	4
jpg	2,422	3,845	4769	6121	6177	5854
html	3	82	17	1764	3116	1947

Total	4 118 180	5 178 174	5 803 225	8 150 729	9 038 016	8 093 261

The first observation is the significant increase in identified accession numbers within supplementary data files, more than doubling over the last five years. This aligns with FAIR principles: by sharing data and including identifiers in the supplementary data files, authors facilitate data integration and cross-referencing across various sources. But the FAIR objective of reusability has its pitfalls when we observe the used file types. In 2022 and 2023, more than 87% of the identified accession numbers were published in Excel files, with a resurgence of the old xls format (6%). 2% of accession numbers are still published in inherently unstructured pdf or Word (docx and doc) documents. Only 8% are published in the more computer-friendly formats csv, txt and xml formats. Finally, beyond using open formats, it is also important to use them correctly: while 99.7% of the xlsx files were extracted correctly, only 93.8% of the csv files were.

It is difficult to analyze the average number of accession numbers per document type, as we know that our approach may underestimate the total. We observed approximately 17 000 files with more than 1000 identified accession numbers, indicating a massive publication of data. 88% of these are Excel files. 2.5% are PDF files; the largest PDF file we observed is named 8972022.f4.pdf, is associated with the article PMC11074859, published in 2024, and contains data on an “Analysis of the RNA transcriptome sequencing data.” It is a 465-page PDF document containing a table with thousands of gene identifiers and GO concepts. Some columns that were too large were spread across several non-consecutive pages, making the data structure impossible to reconstruct. There is no indication that the data were deposited anywhere other than in this supplementary data file. Finally, 2.5% are Word files; the largest Word file we observed is called Table_3.DOCX and is associated with article PMC9279134, published in 2022. It is a 443-page document containing a table with thousands of protein identifiers and GO concepts. The manuscript states that the data have been deposited in FigShare, but it is there in the same Word format. These observations highlight the considerable amount of potentially valuable biological information that is currently housed in supplementary files, often in formats that make machine processing difficult.

## 4 Discussion

A limitation of our study is the focus on Open Access literature, necessitated by our text analysis approach; the generalization of our findings to the broader, including non-Open Access, biomedical literature remains to be fully established. Another limitation is the simplicity of the methods used to identify the names and accession numbers of Global Core Biodata Resources in text. For names, future work could exploit synonyms, abbreviations, or contextual analysis. For accession numbers, a more effective approach than general regular expressions, which can generate noise, could involve searching for exhaustive lists of known accession numbers. These advanced methods would improve recall, potentially at the cost of precision. However, the primary focus of our study is to compare the amount of information about GCBRs contained in manuscripts versus in supplementary data files. As more sophisticated methods would be applied to both, we anticipate that the substantial quantitative differences should not affect our findings qualitatively: with all methods, accession numbers should be massively present in supplementary data files compared to manuscripts. Moreover, it is important to emphasize that the matching strategies employed in this study do not impact the user experience within SIBiLS: a user searching for a specific accession number will still retrieve all documents where that text appears, regardless of whether it was identified as accession number and counted in our analysis.

Therefore, this study highlights the increasing importance of supplementary data files for finding mentions of GCBRs, and the incomplete picture provided by traditional information retrieval methods: search engines in MEDLINE or PMC focus primarily on content within the main manuscript, and may miss a large amount of valuable information. For highly specific topics or curation pipelines, manual examination of supplementary files of retrieved articles should be highly beneficial. However, this can be time-consuming and impractical for large-scale searches. The development of text mining solutions to extract text and make it searchable is therefore crucial for improving their findability. SIBiLS now makes it possible to search for text in a large proportion of supplementary data files, and could significantly improve the efficiency and accuracy of literature searches, particularly for scientists working in data-intensive fields. For example, if a scientist searches for literature published in 2024 containing the term “POLG1” (a human gene), he will retrieve 57 full-text PMC articles containing this term in SIBiLS. Thanks to the supplementary data files collection, he will be able to identify 32 files containing this term, including 15 Excel files, 11 pdfs, and 4 Word files. Of these, 21 belong to articles not present in the PMC results.

The fact that authors are increasingly sharing data in supplementary data files and are increasingly using accession numbers aligns with the FAIR principles. However, these principles are partly undermined by data sharing practices, as authors store a large proportion of accession numbers in Excel files, sometimes in Word files or PDF. While these formats facilitate human readability and accessibility, they pose significant challenges to machine reusability and interoperability. In particular, they typically lack rich metadata describing the data's context, as well as largely adopted standardized structures for data representation. Thus, machines can struggle to consistently identify and extract specific data points in tables (like accession numbers), their associated metadata, and the interactions between them.

Consequently, the management of the research data via the supplementary data files tends to enhance user experience (readability and accessibility) with minimal perceived impact on costs, which may explain why such an approach is still common practice despite the identified challenges for machine processing. To promote FAIR data management, increasingly recommended by publishers like Oxford University Press, alternative approaches include:

- When appropriated, deposition in specialized repositories, such as EMBL/GenBank/DDBJ recommended for nucleic acid sequence information. This approach generally ensures a high level of FAIR-ness. However, the linkage between the article and the deposited dataset can rely only on free text contained in the manuscript, complicating automated integration. This approach is covering an unknown fraction of the data.
- for data without specialized repositories, deposition in general-purpose repositories (e.g. Zenodo, Dryad, Figshare). These repositories typically impose few constraints about metadata completeness and quality. A more stringent approach could be implemented where mandatory capture of rich metadata is implemented. However, concerns may arise that such coercion approach may be misleading both regarding the quality of the captured metadata, as well as the inflation of costs associated with data management in the budget allocated to scientific projects (e.g. Data Management Plans no more mandatory for the Swiss National Foundation's funded projects).

Addressing these challenges would require exploring incentives for comprehensive metadata and developing machine-readable linking mechanisms between publications and their underlying data. It is plausible that a significant portion of supplementary data files is often finalized or even generated after the initial manuscript submission and potentially even after revisions. Implementing data deposition at the time of manuscript submission could be a promising way forward. This early integration would not only facilitate a more rigorous and transparent peer-review process but also proactively encourage authors to produce well-organized and FAIR-compliant depositions, by embedding data preparation in the active experimental phase. Finally, alternative approaches to handling tabular datasets have been proposed (e.g. CSVW at https://www.w3.org/ns/csvw) to add machine-readable metadata describing their structure, semantics, and context. However, they are often considered to be relatively cumbersome—and therefore not cost-effective—for scientists lacking data expertise. Such data management efforts could be complemented by automation tools capable of interpreting table contents, potentially enabling direct use in services such as SIBiLS.

## Supplementary Material

vbaf155_Supplementary_Data

## Data Availability

All extracted data are accessible and searchable as a collection in the BiodiversityPMC platform (https://biodiversitypmc.sibils.org/). The regular expressions and the Python code used for this study have been deposited on https://github.com/sibils/GCBR_match.
