# Supplementary material for: Unlocking the potential of PubMed Central supplementary data files
Source: Bioinform Adv. 2025 Jun 27;5(1):vbaf155. doi: 10.1093/bioadv/vbaf155 (PMC12371329; doi:10.1093/bioadv/vbaf155)
Supplement: vbaf155_Supplementary_Data [file vbaf155_supplementary_data.zip › SIBiLS suppdata Bioinformatics_v4.docx]

*Bioinformatics Advances*, YYYY, 0–0

doi: 10.1093/Bioinformatics Advances/xxxxx

Advance Access Publication Date: DD Month YYYY

Manuscript Category

| *Data and text mining*  Unlocking the potential of PubMed Central Supplementary Data Files  Julien Gobeill^1,2*^, Déborah Caucheteur^1,2^, Alexandre Flament^1,2^, Pierre-André Michel^1,2^, Anaïs Mottaz^1,2^, Emilie Pasche^1,2^ and Patrick Ruch^1,2^  ^1^ SIB Text Mining group, Swiss Institute of Bioinformatics, 1206 Geneva, Switzerland, ^2^ BiTeM group, Information Sciences, HES-SO / HEG Geneva, 1227 Carouge, Switzerland.  *To whom correspondence should be addressed.  Associate Editor: XXXXXXX  Running head : Unlocking the potential of PMC Supp. Data Files  Abstract  **Motivation:** Biocuration workflows often rely on comprehensive literature searches for specific biological enti-ties. However, standard search engines such as MEDLINE and PubMed Central provide an incom-plete picture of the scientific literature because they do not index the increasing amount of valuable information published in supplementary data files. Over two years, we addressed this gap by sys-tematically extracting text from a large proportion (85%) of these files, resulting in 35 million search-able documents. To assess the information gain provided by supplementary data files beyond the manuscripts, we searched both for mentions of dozens of Global Core Biodata Resources (GCBRs), which are fundamental biological databases essential for the life sciences. We searched for men-tions of GCBR names, and accession numbers which uniquely identify biological entities within these resources.  **Results:** The recall gain from using the supplementary data files to search for articles mentioning resource names is 6%. In addition, 97% of all accession numbers identified were published in the supple-mentary data files, highlighting their increasing importance for highly specific topics or curation pipelines. We show that the number of accession numbers published in the supplementary data files is increasing year on year, but that 87% of these are published in Excel files. This format facili-tates human readability and accessibility, but severely limits machine reusability and interoperabil-ity. We therefore discuss alternative and complementary approaches to the publication of research data.  **Availability and implementation:** All extracted data are accessible and searchable as a collection on the BiodiversityPMC platform (https://biodiversitypmc.sibils.org/).  **Keywords: Databases, Text mining, Information retrieval** |
| --- |

# Introduction

MEDLINE and PubMed Central (PMC) are well organized big data, providing access to vast biomedical literature: PMC offers free full-text search and access to research articles, complementing the bibliographic details and abstracts available in MEDLINE (Delamotte 2001). Both collections and their search engines play crucial roles in biocuration workflow for linking biological databases with literature (Bourne 2006), enhanced or complemented by text mining solutions (Neveol 2012, Hirschman 2012). Concerning the comparative utility of ab-stracts and full texts, numerous studies have demonstrated the enhanced information retrieval potential of full-text articles compared to abstracts alone (Lin 2009, Blake 2010, Westergaard 2018).

Yet, search engines in MEDLINE and PubMed Central provide an incomplete picture of the scientific literature. Beyond the abstract and the full-text, publications are often accompanied by so far “dark data”: the supplementary data files, which contain valuable information that support the article but are not included in the core manuscript. These supplementary data files can contain detailed methods, additional results, raw data tables or extended figures (Ramstrand 2020, Boll 2007). But they are highly heterogeneous (images, documents, spread-sheets, code…), and often stored in proprietary formats (e.g. Word, Excel…). This vast amount of valuable information collectively forms a significant portion of scientific knowledge and is critical for scientific progress (Howe 2008, Ferguson 2014). However, the supplemen-tary data files are currently beyond the scope of literature search, as they are not carefully indexed and stored. They become nearly invisi-ble to scientists, and more likely to remain underutilized or eventually lost (Heidorn 2008). Furthermore, concerns have been raised (Greenbaum 2017) about the need to improve the structure of supplementary files to better facilitate the application of the FAIR principles (Wilkinson 2016): findability, accessibility, interoperability and reproducibility.

While the existing literature reveals limited research on this topic, some studies showed that supplementary data files could enhance the impact of information retrieval, by providing valuable information beyond what is available in MEDLINE and PMC (Kafkas 2015, Jimeno Yepes 2014, Naderi 2022, Pasche 2023). However, these studies have been conducted on samples of PMC, and/or focused on a limited number of file types. Recently, BioStudies (Sarkans 2018) serves as a repository that organizes and provides access to supplementary data files associated with life sciences studies, including those from Europe PMC. Yet, BioStudies does not directly extract text from supple-mentary data files, and therefore does not make them findable via a search engine.

During two years, we have systematically downloaded all PMC supplementary data files, and extracted the text from a large proportion of them. Extracted texts were then indexed and made available as a new collection in our Swiss Institute of Bioinformatics Literature Services (SIBiLS), accessible via RESTFUL APIs for searching and fetching, and a Web search engine (Gobeill 2020). To assess the information gain provided by the supplementary data files, we exploited the Global Core Biodata Resources (GCBR), a set of fundamental biological data repositories that are crucial for life science research worldwide (Cook 2023). For the 52 resources included in 2024, we searched for all references to their names in the supplementary data files, and compared them with references in MEDLINE and PMC. We then repeated this work with accession numbers, which are unique and stable identifiers assigned to specific entries within GCBRs, e.g. “GO:0005739” which is the accession number in the Gene Ontology for the concept “mitochondrion”. Matching an accession number within a text strongly suggests the presence of information about a particular biological entity.

In this article, we describe the methods used to extract text from these heterogeneous files, and to recognize GCBR concepts in text. We show that the supplementary data files contain a massive amount of valuable keywords and accession numbers that are not present in the manuscript of the article, and would otherwise be missed by a search in PubMed or PMC. We also show how this data sharing is evolving, and what file formats are being used by authors.

# Methods

The experiments reported in this article were performed in July 2024 using 37.4 million bibliographic references from MEDLINE, 6.2 million manuscripts from the PMC Open Access (OA) set, and their associated supplementary data files. In this section we first present SIBiLS, which already contained the MEDLINE and PMC collections, and into which we have integrated the supplementary data files col-lection. We then describe how we extracted the text from these supplementary data files. Finally, we describe how we searched for GCBR names and accession numbers in all these collections.

## Integration of supplementary data files in SIBiLS

The Swiss Institute of Bioinformatics Literature Services (SIBiLS) is a resource for personalized Information Retrieval of biomedical litera-ture. SIBiLS collects and updates several collections on a daily basis. All documents are parsed and then semantically enriched by automat-ic annotation using about thirty reference controlled vocabularies, such as Medical Subject Headings (MeSH), Open Tree of Life or Drug-Bank. Users can search the collection using the classic SIBiLS Web search engine, or by submitting their own personalized query (using Elastic Query Language) via the RESTFUL APIs. Users can also retrieve a set of documents along with their annotations, in json BioC format, thanks to the fetch API. The Figure 1 illustrates the general workflow of SIBiLS.

MEDLINE and PMC collections are both collected using the National Library of Medicine (NLM) ftp server (ftp.ncbi.nlm.nih.gov). The NLM updates these collections daily by providing tar.gz files containing one xml file for each document added or updated. For the last two years, SIBiLS has also indexed a collection of supplementary data files from a subset of PMC articles that were presumably relevant to genetic variants, obtained using a Boolean search query (Pasche 2023). In parallel, we extracted the text of supplementary data files from the entire PMC collection, which have been fully available in SIBiLS since July 2024. In this collection, each supplementary data file is considered as a document. A unique identifier is created by concatenating the pmcid (PMC article identifier) and the filename (e.g. PMC11065014_media-1.pdf).

## Text extraction from the supplementary data files

For each article in the PMC OA set, a tar.gz archive containing a set of files (both related to the manuscript and the supplementary data files) is available on the NLM ftp server. Among these files, we have excluded the nxml files that contain the manuscript, and whose in-formation is therefore already in the PMC collection. We also excluded pdf files with the same name as the nxml, as this is the pdf version of the manuscript. We processed all jpg files, but we distinguished between figures that are part of the manuscript (their names are indicated in the nxml) and those that are really supplementary data. Finally, we excluded gif images, as they are either miniature versions of a figure in jpg (for Web printing) or equation symbols. This left 36.2M files for 6.2M articles, with more than 1,200 different file types.

We then used several methods to extract the text from different types of files:

1. for jpg files, we used a local API (https://ocrweb.text-analytics.ch/) based on Tesseract (Smith 2007), a popular Optical Characters Recognition (OCR) software with proven accuracy (Tafti 2016). Our API optimizes the OCR by applying rotations to the image to enable vertical text to be recognized.
2. for pdf, we used the Python module PyPDF2 (https://pypi.org/project/PyPDF2/). This module extracts text stored as actual characters (i.e. searchable and selectable).
3. for tables (xlsx, xls and csv) we used a local extractor developed by (Naderi 2022) based on the Python module pandas (McKinney 2011).
4. for Word documents (doc, docx), we used the Python modules textract and doc2txt (https://textract.readthedocs.io/en/stable/, https://pypi.org/project/docx2txt/).
5. for html and xml, we extracted the text using the Python module BeautifulSoup (https://pypi.org/project/beautifulsoup4/).

It took two years to download and process all the supplementary data, with parallel programs. The Table 1 shows the twenty most com-mon file types in the supplementary data files, which represents 98% of all the files.

A further 24,491,367 jpg files are figures that are part of the manuscript. They are not included in the previous table, but we have also processed them to include their text in our supplementary data files index (as their text is not indexed and therefore not searchable in the PMC collection).

In terms of supplementary data files alone, we thus extracted text from 84.6% of the files. The initial design of our text extraction pipe-line was based on the assumption that jpg would be the dominant format for figures and images in supplementary data files; however, we later observed that tiff and png formats represented a non-negligible 7% of the image data, which were not processed. The zip files (2.64%) are currently being processed but were not yet available at the time of the study. Finally, more than 3% of the files are multimedia videos (mp4, avi, mov, flv, webm), which seem more difficult for us to process.

Extraction success rates are nearly 100%, except for older Microsoft doc (97.4%) and xls (95.7%) formats, where some files present is-sues even when opened manually with the correct software, and for csv files (93.8%), which may contain formatting errors or problematic encoding. For jpg, 23% of successfully extracted files have no extracted text. For the rest, output quality may be low depending on the image resolution. Additionally, while text from doc and pdf files is extracted, some of these files consist of scanned images, and OCR is not applied to them.

## Search for references to Global Core Biodata Resources

In July 2024 there were 52 resources listed as GCBR. We took the 52 names and searched for them in SIBiLS, first in the MEDLINE col-lection (title + abstract), then in the PMC collection (full text), and finally in the supplementary data files collection (text extracted from the files).

The resources names were searched thanks to regular expressions using lowercasing and word boundaries, ensuring for example that Rhea is not recognized in the word “spearhead” (>3,000 results in PMC). We considered only articles published from 2000. For MEDLINE, we used the pmcid as the unique identifier of the article when it was available, to avoid counting the same article twice if the mention was in both the abstract (MEDLINE and PMC) and the full text (PMC). For 72% of the retrieved MEDLINE citations, the article was available in PMC. For supplementary data files, we have also used the article pmcid, to avoid counting the same article twice if the mention was in both the abstract or full text (MEDLINE and PMC) and the supplementary data files. Four resources were excluded from the results after a manual scan of a sample of 1,000 sentences containing a mention, since they caused between 50% and 75% of False Positives: BRENDA and SILVA (which were confused with first names or surnames), CATH (confused with the abbreviation of catheter, e.g. “cath lab”), and STRING (confused with the common noun “string”).

For the accession numbers, we also used regular expressions, collected in the Identifiers.org Central Registry, a service which provides a centralized directory of Compact Identifiers for biodata resources (https://registry.identifiers.org/). Only 30 of the 52 resources were found in the registry. These are listed in Table 3. For each resource, we exploited its “Local Unique Identifier pattern”, a regular expression (e.g. r”GO:\d{7]” for the Gene Ontology). Pre-experiments showed that many common nouns were identified as accession numbers because certain regular expressions were too general, such as r"[23456789BCDFGHJKLMNPQRSTVWXYZ]{1,6}” for Catalog Of Life. The same problem occurred with UniProt identifiers. We then decided to force the presence of the resource (e.g. “col:4QHKG”, “UniProt:Q8VRN4”) for those problematic regular expressions. This probably led to missed identifiers, but solved the problem of False Positives in the sam-ples. We also used lowercasing for regular expressions.

We used different counting strategies for resource names and accession numbers, depending on our specific objectives. For resource names, we were interested in the number of articles that cited a resource. Therefore, we counted a citation only once per article, regardless of whether it appeared in the manuscript, supplementary data files, or both. For accession numbers, however, our aim was to quantify the number of additional mentions in the supplementary data files. Therefore, we counted each identified accession number to directly com-pare its prevalence between the manuscript and the supplementary data files. Articles in MEDLINE and PMC include both title and abstract, but we were careful not to count accession numbers twice.

It is crucial to note that the primary focus of this study is not to exhaustively identify every mention of GCBRs, but rather to compare the relative amount of information within supplementary data files against within manuscripts. For this purpose, we assume that we can rely on simple methodological approach, prioritizing high precision (minimizing false positives) over maximal recall (capturing all possible in-stances).

# Results

## The growing amount of available supplementary data files

We first observed the evolution of the number of supplementary data files according to the publication year, which is illustrated in the Figure 2. The number of open access articles is increasing year on year, and the number of articles with supplementary data files has risen from around 40% in the early 2000s to around 80% in the 2020s. This suggests that authors are increasingly including supplementary data to support their findings, possibly due to journal requirements to promote FAIRness, or due to the availability of data offered by the com-puter generation of large datasets. In the early 2020s, there is a significant rise in the number of OA articles, likely due to the impact of Covid-19 on publication volume, as well as a shift in the publishing landscape driven by Open Access mandates from funding institutions. The reasons for the concomitant slight decrease in the average number of supplementary files have yet to be explained. Finally, the smaller number of articles available for 2023 may be explained by the fact that some are still under publication embargo in mid-2024.

## References to GCBR names in supplementary data files

We then report in Table 2 the number of articles with mentions of GCBR names in the three collections available: first by searching MEDLINE only, then MEDLINE and PMC, then MEDLINE and PMC and supplementary data files (abbreviated as suppdata in the follow-ing tables). Finally, we looked at the benefits of using supplementary data files to find articles that mentioned a resource, compared to searching MEDLINE and PMC alone.

Overall, the recall gain using the supplementary data files is 6%, with notable values for Bgee (+28%), InterPro (+25%) and EuropePMC (+24%). In this work, we have taken the names of the resources as given by the Global Biodata Coalition. This led to False Positives, the main sources of which were discarded, but certainly also to False Negatives. For example, “Orphadata science” had no match, whereas “Orphadata” returns over 1,000 results in PMC.

Authors may therefore use a resource in their work without mentioning it in the manuscript, but only in the supplementary data files. The impact of these resources is therefore greater than what can be measured by a search in MEDLINE or PMC alone.

## References to GCBR accession numbers in supplementary data files

We then report in Table 3 the total number of accession numbers found in the three collections, using the regular expressions collected from identifiers.org for 30 resources: first by considering MEDLINE only, then MEDLINE and PMC, then MEDLINE and PMC and supplementary data files. Finally, we looked at the percentage of accession numbers that were only present in the supplementary data files.

97% of all mentions of accession numbers are found in the supplementary data files. While the recall gain was significant but modest for resources names (+6%), here the number of retrieved documents is multiplied by 33, rising from 1.9 to 62.9 million. The most frequent mentions come from the Gene Ontology, with nearly 30 million references, and Human Protein Atlas, with 18 million. We suspect that the number of UniProt accession numbers contained in the supplementary data files is actually much higher, but it would have been impossible to identify them correctly without the restrictions we imposed by forcing the resource name beforehand (e.g. “UniProt:Q8VRN4”). The only resource without found mentions is Clinical Interpretation of Variants in Cancer (r”civic.vid:\d+”).

It is therefore certain that by searching MEDLINE and PMC alone, a scientist looking for literature on a particular accession number will miss the largest part of references.

For assessing the quality of extracted accession numbers and proving that our assertions were right, we randomly selected 1,000 of them, and checked if they were correct, i.e. if they existed in the related biodata resource. The results are presented in Table 4.

These results show that the regular expressions provided by identifiers.org are extremely accurate (99.9%). The only spotted error was an incorrect Bgee accession number (FBgn003202) which was actually FBgn0032029 in the original file (a nine-page table in doc format) and was badly extracted. Such tables in Word documents may be challenging to parse, due to lack of clear borders, merged cells, or inconsistent spacing.

## Evolution by publication years and file types

Finally, we observed how the number of accession numbers has evolved according to the publication year, and the type of file used, and reported the results in Table 5.

The first observation is the significant increase in identified accession numbers within supplementary data files, more than doubling over the last five years. This aligns with FAIR principles: by sharing data and including identifiers in the supplementary data files, authors facilitate data integration and cross-referencing across various sources. But the FAIR objective of reusability has its pitfalls when we observe the used file types. In 2022 and 2023, more than 87% of the identified accession numbers were published in Excel files, with a resurgence of the old xls format (6%). 2% of accession numbers are still published in inherently unstructured pdf or Word (docx and doc) documents. Only 8% are published in the more computer-friendly formats csv, txt and xml formats. Finally, beyond using open formats, it is also important to use them correctly: while 99.7% of the xlsx files were extracted correctly, only 93.8% of the csv files were.

It is difficult to analyze the average number of accession numbers per document type, as we know that our approach may underestimate the total. We observed approximately 17,000 files with more than 1,000 identified accession numbers, indicating a massive publication of data. 88% of these are Excel files. 2.5% are pdf files; the largest pdf file we observed is named 8972022.f4.pdf, is associated with the article PMC11074859, published in 2024, and contains data on an “Analysis of the RNA transcriptome sequencing data”. It is a 465-page pdf document containing a table with thousands of gene identifiers and GO concepts. Some columns that were too large were spread across several non-consecutive pages, making the data structure impossible to reconstruct. There is no indication that the data were deposited anywhere other than in this supplementary data file. Finally, 2.5% are Word files; the largest Word file we observed is called Table_3.DOCX and is associated with article PMC9279134, published in 2022. It is a 443 page document containing a table with thousands of protein identifiers and GO concepts. The manuscript states that the data has been deposited in FigShare, but it is there in the same Word format. These observations highlight the considerable amount of potentially valuable biological information that is currently housed in supplementary files, often in formats that make machine processing difficult.

# Discussion

A limitation of our study is the focus on Open Access literature, necessitated by our text analysis approach; the generalization of our findings to the broader, including non-Open Access, biomedical literature remains to be fully established. Another limitation is the simplicity of the methods used to identify the names and accession numbers of Global Core Biodata Resources in text. For names, future work could exploit synonyms, abbreviations, or contextual analysis. For accession numbers, a more effective approach than general regular expressions, which can generate noise, could involve searching for exhaustive lists of known accession numbers. These advanced methods would improve recall, potentially at the cost of precision. However, the primary focus of our study is to compare the amount of information about GCBRs contained in manuscripts versus in supplementary data files. As more sophisticated methods would be applied to both, we anticipate that the substantial quantitative differences should not affect our findings qualitatively: with all methods, accession numbers should be massively present in supplementary data files compared to manuscripts. Moreover, it is important to emphasize that the matching strategies employed in this study do not impact the user experience within SIBiLS: a user searching for a specific accession number will still retrieve all documents where that text appears, regardless of whether it was identified as accession number and counted in our analysis.

Therefore, this study highlights the increasing importance of supplementary data files for finding mentions of GCBRs, and the incomplete picture provided by traditional information retrieval methods: search engines in MEDLINE or PMC focus primarily on content within the main manuscript, and may miss a large amount of valuable information. For highly specific topics or curation pipelines, manual examination of supplementary files of retrieved articles should be highly beneficial. However, this can be time-consuming and impractical for large-scale searches. The development of text mining solutions to extract text and make it searchable is therefore crucial for improving their findability. SIBiLS now makes it possible to search for text in a large proportion of supplementary data files, and could significantly improve the efficiency and accuracy of literature searches, particularly for scientists working in data-intensive fields. For example, if a scientist searches for literature published in 2024 containing the term “POLG1” (a human gene), he will retrieve 61 full-text PMC articles containing this term in SIBiLS. Thanks to the supplementary data files collection, he will be able to identify 221 files containing this term, including 94 Excel files, 63 pdfs, and 51 Word files. Of these, 216 belong to articles not present in the PMC results.

The fact that authors are increasingly sharing data in supplementary data files and are increasingly using accession numbers aligns with the FAIR principles. However, these principles are partly undermined by data sharing practices, as authors store a large proportion of accession numbers in Excel files, sometimes in Word files or pdf. While these formats facilitate human readability and accessibility, they pose significant challenges to machine reusability and interoperability. In particular, they typically lack rich metadata describing the data's context, as well as largely adopted standardized structures for data representation. Thus, machines can struggle to consistently identify and extract specific data points in tables (like accession numbers), their associated metadata, and the interactions between them.

Consequently, the management of the research data via the supplementary data files tend to enhance user experience (readability and accessibility) with minimal perceived impact on costs, which may explain why such an approach is still common practice despite the identified challenges for machine processing. To promote FAIR data management, increasingly recommended by publishers like Oxford University Press, alternative approaches include:

1. when appropriated, deposition in specialized repositories, such as EMBL/GenBank/DDBJ recommended for nucleic acid sequence information. This approach generally ensures a high level of FAIR-ness. However, the linkage between the article and the deposited dataset can rely only on free text contained in the manuscript, complicating automated integration. This approach is covering an unknown fraction of the data.
2. for data without specialized repositories, deposition in general-purpose repositories (e.g. Zenodo, Dryad, Figshare). These repositories typically impose few constraints about metadata completeness and quality. A more stringent approach could be implemented where mandatory capture of rich metadata is implemented. However, concerns may arise that such coercion approach may be misleading both regarding the quality of the captured metadata, as well as the inflation of costs associated with data management in the budget allocated to scientific projects (e.g. Data Management Plans no more mandatory for the Swiss National Fundation's funded projects).

Addressing these challenges would require exploring incentives for comprehensive metadata and developing machine-readable linking mechanisms between publications and their underlying data. It is plausible that a significant portion of supplementary data files is often finalized or even generated after the initial manuscript submission and potentially even after revisions. Implementing data deposition at the time of manuscript submission could be a promising way forward. This early integration would not only facilitate a more rigorous and transparent peer-review process, but also proactively encourage authors to produce well-organized and FAIR-compliant depositions, by embedding data preparation in the active experimental phase. Finally, alternative approaches to handling tabular datasets have been proposed (e.g. CSVW at https://www.w3.org/ns/csvw) to add machine-readable metadata describing their structure, semantics and context. However, they are often considered to be relatively cumbersome - and therefore not cost-effective - for scientists lacking data expertise. Such data management efforts could be complemented by automation tools capable of interpreting table contents, potentially enabling direct use in services such as SIBiLS.

Acknowledgements

We acknowledge the members of the Elixir Data Platform, Work Package 4 “Scalable curation support from the long tail of biological data”.

**Conflict of interest**

None declared.

**Funding**

This work has been supported by the Elixir Data Platform.

**Data availability**

All extracted data are accessible and searchable as a collection in the BiodiversityPMC platform (https://biodiversitypmc.sibils.org/).

The regular expressions and the Python code used for this study have been deposited on https://github.com/sibils/GCBR_match.

References

Blake, C. (2010). Beyond genes, proteins, and abstracts: Identifying scientific claims from full-text biomedical articles. Journal of biomedical informatics, 43(2), 173-189.

Boll, S. (2007). Share it, reveal it, reuse it, and push multimedia into a new decade. IEEE MultiMedia, 14(4), 14-19.

Bourne, P. E., & McEntyre, J. (2006). Biocurators: contributors to the world of science. PLoS computational biology, 2(10), e142.

Cook, C., & Cochrane, G. (2023). The Global Biodata Coalition: Towards a sustainable biodata infrastructure. Biodiversity Information Science and Standards, (e93453).

Delamothe, T., & Smith, R. (2001). PubMed Central: creating an Aladdin's cave of ideas: We have seen the future, and it works. BMJ, 322(7277), 1-2.

Ferguson, A. R., Nielson, J. L., Cragin, M. H., Bandrowski, A. E., & Martone, M. E. (2014). Big data from small data: data-sharing in the long tail of neuroscience. Nature neuroscience, 17(11), 1442-1447.

Gobeill, J., Caucheteur, D., Michel, P. A., Mottin, L., Pasche, E., & Ruch, P. (2020). SIB Literature Services: RESTful customizable search engines in biomedical literature, enriched with automatically mapped biomedical concepts. Nucleic acids research, 48(W1), W12-W16.

Greenbaum, D., Rozowsky, J., Stodden, V., & Gerstein, M. (2017). Structuring supplemental materials in support of reproducibility. Genome Biology, 18, 1-5.

Heidorn, P. B. (2008). Shedding light on the dark data in the long tail of science. Library trends, 57(2), 280-299.

Hirschman, L., Burns, G. A., Krallinger, M., Arighi, C., Cohen, K. B., Valencia, A., ... & Winter, A. G. (2012). Text mining for the biocuration workflow. Database, 2012.

Howe, D., Costanzo, M., Fey, P., Gojobori, T., Hannick, L., Hide, W., ... & Yon Rhee, S. (2008). The future of biocuration. Nature, 455(7209), 47-50.

Jimeno Yepes, A., & Verspoor, K. (2014). Literature mining of genetic variants for curation: quantifying the importance of supplementary material. Database, 2014, bau003.

Kafkas, Ş., Kim, J. H., Pi, X., & McEntyre, J. R. (2015). Database citation in supplementary data linked to Europe PubMed Central full text biomedical articles. Journal of Biomedical Semantics, 6, 1-7.

Lin, J. (2009). Is searching full text more effective than searching abstracts?. BMC bioinformatics, 10, 1-15.

McKinney, W. (2011). pandas: a foundational Python library for data analysis and statistics. Python for high performance and scientific computing, 14(9), 1-9.

Naderi, N., Mottaz, A., Teodoro, D., & Ruch, P. (2022). Analyzing the information content of text-based files in supplementary materials of biomedical literature. In Challenges of Trustable AI and Added-Value on Health (pp. 876-877). IOS Press.

Névéol, A., Wilbur, W. J., & Lu, Z. (2012). Improving links between literature and biological data with text mining: a case study with GEO, PDB and MEDLINE. Data-base, 2012, bas026.

Pasche, E., Mottaz, A., Gobeill, J., Michel, P. A., Caucheteur, D., Naderi, N., & Ruch, P. (2023). Assessing the use of supplementary materials to improve genomic variant discovery. Database, 2023, baad017.

Ramstrand, N., Fatone, S., Dillon, M. P., & Hafner, B. J. (2020). Sharing research data. Prosthetics and orthotics international, 44(2), 49-51.

Sarkans, U., Gostev, M., Athar, A., Behrangi, E., Melnichuk, O., Ali, A., ... & McEntyre, J. (2018). The BioStudies database—one stop shop for all data supporting a life sciences study. Nucleic acids research, 46(D1), D1266-D1270.

Smith, R. (2007, September). An overview of the Tesseract OCR engine. In Ninth international conference on document analysis and recognition (ICDAR 2007) (Vol. 2, pp. 629-633). IEEE.

Tafti, A. P., Baghaie, A., Assefi, M., Arabnia, H. R., Yu, Z., & Peissig, P. (2016). OCR as a service: an experimental evaluation of Google Docs OCR, Tesseract, ABBYY FineReader, and Transym. In Advances in Visual Computing: 12th International Symposium, ISVC 2016, Las Vegas, NV, USA, December 12-14, 2016, Proceedings, Part I 12 (pp. 735-746). Springer International Publishing.

Westergaard, D., Stærfeldt, H. H., Tønsberg, C., Jensen, L. J., & Brunak, S. (2018). A comprehensive and quantitative comparison of text-mining in 15 million full-text articles versus their corresponding abstracts. PLoS computational biology, 14(2), e1005962.

Wilkinson, M. D., Dumontier, M., Aalbersberg, I. J., Appleton, G., Axton, M., Baak, A., ... & Mons, B. (2016). The FAIR Guiding Principles for scientific data management and stewardship. Scientific data, 3(1), 1-9.
